# Disengagement and reintegration as psychosocial capacity-building after violent extremism

**DOI:** 10.3389/fpsyt.2026.1814567

**Published:** 2026-10-16

**Authors:** Deepti Ramaswamy

**Affiliations:** Department of Psychology, The Open University, Milton Keynes, United Kingdom

**Keywords:** disengagement, emotional regulation, epistemic trust, moral agency, narrative identity, reintegration, trauma-informed care, violent extremism

## Abstract

Efforts to address violent extremism increasingly acknowledge the relevance of mental health, yet psychological factors are still often treated as background vulnerabilities rather than as active processes shaping whether reintegration can be sustained over time. This paper offers a conceptual, theory-generating account of how therapeutic engagement supports the consolidation of disengagement and the conditions for sustainable reintegration. All participants had already disengaged before starting therapy, so the paper is not an evaluation of a specific therapy’s effectiveness in supporting disengagement; instead, it traces the psychosocial mechanisms through which change unfolds over time. Drawing on longitudinal narrative interviews with six individuals, the analysis traces changes in emotional regulation, identity, trust in others, and moral reasoning over time. All six had disengaged, for a minimum of two years, from far-right or Islamist extremist or extremist-adjacent environments, or from the incel milieu. They subsequently took part in trauma-informed therapeutic work delivered individually and remotely over approximately nine to twelve months. Participants described therapeutic engagement as supporting emotional safeness and regulation, and their accounts point to an accompanying capacity to revisit past involvement without collapse, integrate responsibility into more coherent self-narratives, and move away from rigid, polarised ways of relating to others. These psychosocial shifts shaped participants’ capacity to engage with everyday social, practical, and civic domains. However, the durability of these changes depended on external conditions, such as stability, social recognition, and access to meaningful roles. Where those conditions were absent, exclusion or precarity could undermine even real psychosocial gains. The study reframes post-exit reintegration as a relational and developmental process of psychosocial capacity-building, in which therapeutic engagement is one enabling condition among others.

## 1 Introduction

Efforts to prevent and address violent extremism have increasingly acknowledged the relevance of mental health and psychological vulnerability in pathways into and out of extremist involvement (1, pp. 4–7). Across the academic literature, factors such as trauma exposure, emotional dysregulation, and adverse developmental experiences are frequently cited in relation to radicalisation, disengagement, and reintegration (e.g., 2, pp. 12–13; 3, pp. 1–27; 4, pp. 9–15; 5, pp. 8–11). This body of work builds on a substantial qualitative tradition of first-hand engagement with extremists and former extremists themselves (6–9). Despite this growing recognition of mental health’s relevance, however, there remains limited conceptual and empirical clarity regarding how these processes actively shape disengagement and reintegration beyond treating them as background vulnerabilities or generic risk factors.

This limitation is particularly visible in post-disengagement contexts. Recent work has noted that reintegration following extremist disengagement is often uneven, protracted, and fragile, and is poorly captured by models that focus primarily on ideological change, programme completion, or formal indicators of compliance (10, pp. 421–424). Individuals may disengage ideologically and receive practical support, yet continue to struggle to sustain social participation, trust in others, or a coherent sense of self.

Without attention to the psychosocial mechanisms through which emotional regulation, epistemic trust, and narrative coherence develop, it remains difficult to explain why disengagement sometimes consolidates into sustained reintegration. In this sense, consolidation refers to change becoming sufficiently stable to be carried beyond the point of exit. In other cases, disengagement falters despite apparent progress.

Early and chronic relational adversity can undermine the development of a coherent self-narrative by disrupting experiences of emotional containment, recognition, and continuity, leaving individuals with fewer opportunities to integrate experience over time into a stable sense of self. Where caregiving is inconsistent or emotionally unpredictable, a stable, internalised sense of self may not develop (11, pp. 140–152). Instead, a child’s sense of self can become contingent on the caregiver’s judgement of them in the moment, good when the caregiver is in a good mood, worthless when they are not, rather than continuous across time and independent of the other person’s state. Yet these developmental vulnerabilities are rarely reflected in how post-exit support is designed. At the same time, many contemporary intervention frameworks prioritise practical, problem-solving approaches such as housing, employment, education, or social support, or position ideological change as the primary lever of disengagement. In this context, social support typically refers to the availability of external resources or services, rather than to the relational and psychological capacity required to trust, make use of, and remain engaged with such support over time. As a result, support may be formally present yet functionally inaccessible when individuals are unable to experience others as safe, reliable, or responsive.

While such measures may be necessary, they often overlook the extent to which individuals leaving extremist or extremist-adjacent environments continue to operate in survival-based modes of functioning (12, pp. 13–17). Survival-based modes of functioning refer to patterns of heightened threat sensitivity, emotional reactivity or numbing, and defensive self-reliance shaped by chronic adversity, which prioritise short-term safety over reflection, trust, or integration. These modes are frequently characterised by affective dysregulation, epistemic mistrust, and fragmented self-narratives, even after ideological identities have begun to loosen.

Where such internal processes remain unaddressed, practical stability alone is unlikely to support durable reintegration (13, pp. 117–145). The implication is not that practical assistance is unnecessary, but that its effectiveness depends on whether individuals have developed sufficient emotional regulation, epistemic trust, and narrative coherence to make use of such support as meaningful, rather than overwhelming or misaligned.

Together, these dynamics point to a more specific gap: limited empirical clarity about how psychosocial capacities such as emotional regulation, narrative coherence, epistemic trust, and moral agency develop over time within relational or therapeutic contexts, and why they matter beyond the point of exit. Building on this diagnosis, this paper examines how therapeutic engagement, supports the consolidation of disengagement, once it has already occurred, and the conditions for sustainable reintegration, through a psychosocial and narrative analysis of former extremists.

For clarity, several terms are used consistently throughout this paper. Epistemic trust refers to the capacity to experience another person as accurately understanding one’s own mind, feelings, and perspective, and, on that basis, to experience the information, guidance, and support they offer as relevant, benign, and worth taking in, rather than as threatening, manipulative, or requiring defensive rejection. Narrative coherence refers to the capacity to organise past, present, and anticipated future experiences into a self-narrative that is continuous, liveable, and able to accommodate responsibility without collapse or defensive fragmentation. Emotional regulation refers to the capacity to modulate the intensity and duration of affective states without recourse to avoidance, suppression, dissociation, or unhelpful coping behaviours. Therapeutic engagement and psychosocial capacity-building, the paper’s central constructs, are defined in full below.

Psychosocial capacity is used in this paper as an integrative heuristic rather than a validated psychometric construct. It denotes the developed ability to regulate affect, sustain coherent self-narratives, exercise moral agency, and experience others as trustworthy sources of understanding and support (epistemic trust). These four capacities are treated as interrelated capacities that contribute to reintegration stability, rather than as a single measurable trait. Psychosocial capacity is distinct from adjacent concepts. Unlike resilience, which describes the capacity to withstand or recover from adversity without necessarily changing how a person relates to others or to themselves, psychosocial capacity concerns whether specific developmental capacities, affect regulation, narrative coherence, epistemic trust, and moral agency, are built over time. Unlike psychological flexibility, which describes moment-to-moment adaptability of behaviour in the service of values (14, pp. 180–198), psychosocial capacity concerns whether the underlying conditions for that adaptability, a coherent self-narrative, a tolerable relationship to one’s own affect, trust in others, are present. Someone can be highly resilient, in the sense of continuing to function despite adversity, and still lack the narrative coherence or epistemic trust needed to experience a new relationship, a job, or a community as safe enough to remain in.

The central research problem is therefore not whether therapy functions as a discrete corrective intervention, but how therapeutic engagement supports the development of these four capacities, and under what conditions such change can be sustained beyond the therapeutic setting.

Therapeutic engagement is defined here as sustained, relationally held psychological work conducted within a trauma-informed framework, in which emotional safety, continuity, and collaborative meaning-making provide conditions for reflection, regulation, and narrative integration over time. This is the author’s own operationalisation, developed to capture qualities participants described as important. It draws on established trauma-informed and relational traditions (15, pp. 599–640; 16, pp. 133–154) rather than referring to a single standardised construct with an existing measure.

Adopting a psychosocial and narrative perspective, the paper frames disengagement and reintegration as relational and developmental processes that unfold over time, extending accounts that have focused primarily on ideological trajectories, structural conditions, or programmatic interventions (17, pp. 130–132). By tracing longitudinal changes within a sustained therapeutic context, the analysis proposes an interpretation of the mechanisms through which emotional regulation, narrative coherence, epistemic trust, and moral agency develop in post-exit contexts, and how these capacities shape individuals’ ability to engage with wider environments. In doing so, the paper advances an analytic account of psychosocial capacity-building rather than claims about prevalence, representativeness, or typical trajectories. It draws on the narrative accounts of the six participants who took part in the therapeutic phase, situating their trajectories in relation to the wider 10-person sample from which they are drawn.

## 2 Materials and methods

### 2.1 Study design

This paper draws on qualitative data from a longitudinal narrative research project, conducted as part of a wider doctoral study examining disengagement and reintegration trajectories among individuals formerly involved in extremist or extremist-adjacent environments. These interviews examined how participants made sense of past involvement, disengagement, and subsequent attempts at reintegration over time.

Of the ten participants in the wider study, six opted into an additional phase of trauma-informed therapeutic work; this paper focuses on those six, tracing the psychosocial processes that unfolded over time within a sustained relational context. Participants’ narrative accounts are the primary analytic material.

A dual-role design was employed in which the researcher also acted as the treating therapist for participants who opted into therapeutic engagement. The researcher is a qualified psychotherapist with clinical experience working with trauma, and an EMDR consultant; therapeutic work drew on principles from Acceptance and Commitment Therapy and Compassion-Focused Therapy alongside this core EMDR expertise. Therapeutic engagement was not treated as an intervention to be evaluated, but as a relational context within which emotional regulation, narrative organisation, and moral reflection could be observed over time, particularly in contexts characterised by epistemic mistrust and defensive self-reliance. Comparable practitioner–researcher designs have been used in psychotherapy and psychosocial research to examine processes that are difficult to access through interview methods alone (18, pp. 1–3; 19, pp. 15–16). The ethical and epistemic implications of this design are addressed directly in the following section.

### 2.2 Reflexivity and the dual role

The dual role of therapist and narrative interviewer was adopted because processes of emotional regulation, narrative repair, and moral reappraisal are often difficult to access through interview methods alone (18, pp. 1–3; 19, pp. 15–16), particularly among people shaped by chronic mistrust of professionals and institutions (20, pp. 119–128). This proximity carries risks that bear directly on how the findings should be read: participants’ narratives are likely to have been shaped, in part, by the therapeutic relationship itself, and the power asymmetry inherent in that relationship could plausibly bias moral framing or narrative coherence toward outcomes the researcher, as therapist, hoped to see.

Several safeguards were used to manage this risk rather than to claim it away. Role-signalling made explicit, at the outset of each contact, whether a session was constituted as therapy or as research, so that participants retained a clear sense of which relationship they were in (19, pp. 328–346; 21, pp. 76–89). Only material generated within scheduled narrative interviews was treated as analytic data; clinical process notes were retained solely for supervision and continuity of care and were excluded from coding. Consent was revisited on an ongoing basis rather than treated as a single event, and participants could withdraw from either the therapeutic or the research component independently of the other.

To guard against confirmation bias, analytic claims were grounded explicitly in what participants said within transcribed interviews rather than in the researcher’s clinical impressions, checked against the interview record rather than clinical memory, and refined through supervision and peer review. The analysis therefore distinguishes, as far as is possible within a relationally embedded design, between therapeutic change (shifts participants described in their internal experience and everyday functioning) and narrative performance (accounts shaped primarily by a wish to satisfy the researcher or to present a coherent recovery story to a trusted clinician). Where an account appeared to track the pacing of the therapeutic relationship more closely than the substance of a described capacity, for example a sudden, unprompted expression of resolution or gratitude unsupported by concrete examples of changed functioning between interviews, this was treated cautiously in analysis rather than presented as consolidated change.

These safeguards address one version of this risk: that participants may have shaped their accounts to satisfy the researcher, or that the researcher may have been more attentive to change-consistent material. A further, more fundamental version remains. After many months of shared therapeutic work, the very categories participants used to describe their experience, emotional regulation, responsibility, trust, moral reasoning, may themselves have been shaped by the language and ways of understanding developed within therapy. This is a different and less tractable problem than narrative performance, since it cannot be resolved by checking accounts against the interview record: the interview record is itself part of what was co-constructed. The safeguards above reduce the risk that participants misrepresented their experience to please the researcher, but they cannot fully separate participants’ self-understanding from the relational and conceptual frame within which that self-understanding developed. This is treated here as a feature of the design that further safeguards could not resolve, and it is reflected in the caution with which mechanism and sequence claims are framed throughout this paper.

At the same time, the dual role is not treated solely as a risk to be contained. The relational continuity, safety, and containment the design required to be conducted responsibly are themselves part of what this paper argues is necessary for reintegration to consolidate. What became possible for participants within this relationship, and what remained difficult or absent in the environments they encountered beyond it, is treated as analytically informative rather than as a confound to be explained away. This containment work was not one-directional: sustained engagement with participants’ accounts of trauma and past harm carries a recognised emotional toll on the researcher as well, consistent with broader accounts of the emotional labour involved in extremism fieldwork (22, pp. 1–22). This was managed through regular clinical and research supervision, the same principle of sustained relational support the analysis identifies as necessary for participants themselves.

### 2.3 Participants

Ten self-disengaged former extremists were recruited through a combination of mediated access and snowball sampling, using intermediaries with pre-existing relational credibility in post-exit networks, including practitioners, community contacts, and individuals with lived experience of extremist involvement, rather than institutional referral, public advertisement, or diagnostic screening. Public or institutionally mediated recruitment was assessed as unlikely to generate genuinely voluntary engagement from a population wary of state or media involvement (23, pp. 193–211).

Eligibility required that participants had disengaged from a far-right or Islamist extremist or extremist-adjacent environment, or from the incel milieu, for a minimum of two years prior to recruitment. They also needed to be engaged in, or clearly committed to, counter-extremism or counter-violence activity at the time of recruitment, including mentoring, advocacy, preventative education, or participation in structured exit organisations such as Life After Hate. No formal screening for current risk was conducted beyond this; suitability for the relational and emotional demands of the study was instead assessed jointly with participants and intermediaries on a case-by-case basis, consistent with the study’s trauma-informed orientation.

This process produced a sample of eight men and two women, ranging in age from their twenties to their fifties, with six participants from far-right milieus, three from Islamist contexts, and one from the incel milieu, a distribution reflecting recruitment realities in a hard-to-access population. “Extremist” and “extremist-adjacent” refer to involvement in grievance-based ideological ecosystems characterised by rigid in-group/out-group cognition, identity fusion, and, in some cases, endorsement or fantasy of violence (24, pp. 565–587; 25, pp. 1–29; 26, pp. 336–351), whether that involvement took the form of formal organisational membership or independent identification with a movement’s ideology.

Unlike far-right or Islamist milieus, incel involvement does not typically involve formal group membership, so the “extremist”/”extremist-adjacent” distinction applied elsewhere in this sample does not straightforwardly apply here. The relevant debate instead concerns whether ideological identification with incel communities constitutes extremist involvement at all, given the movement’s diffuse, largely online, and non-organisational character (27). This paper follows the scholarship cited above in treating classification as a matter of ideological content and embeddedness within a wider ecosystem of grievance-based, dehumanising belief, rather than formal group affiliation or actor type, and in treating misogynist incel communities as warranting analysis within a violent extremism framework on this basis. The participant from this milieu is included accordingly.

Not everyone approached joined the study. Approximately six further individuals were proposed or approached during recruitment but did not join. Reasons included discomfort with the researcher’s positionality, protectiveness on the part of intermediaries, a preference for a public ‘former’ identity over reflective engagement, and, in one case, an ethical concern that made participation inappropriate for the study.

Of the ten who did join the study, six participants (P3, P4, P5, P7, P8, and P9) also engaged in the therapeutic phase analysed here. The remaining four did not continue into this phase, citing reasons that included prior completion of trauma-focused therapy elsewhere, a competing acute crisis, or a preference to share their story without therapeutic processing. These four are discussed here, rather than omitted, because their non-participation is analytically relevant: it indicates that entry into the therapeutic phase was shaped by each participant’s own circumstances, rather than simply by interest or need. This means the six participants who did continue were not a random subset of the ten. Whatever the specific reason in each case, the six who did enter therapy may share characteristics, such as greater stability, motivation, or disposition toward reflective engagement, that distinguish them from those who did not, and this raises a legitimate selection question, addressed further under Limitations, about what that implies for how the findings should be read. The narrative material of these four participants formed part of the wider doctoral study from which this paper is drawn but falls outside the scope of the present analysis.

Across the six participants analysed here and the wider sample of ten, individual involvement in these environments ranged from roughly one year to over a decade, predominantly in the UK and North America. Roles varied in form and seriousness and included recruitment and outreach, fundraising, production and amplification of ideological content online, enforcement of internal ideological conformity, and, for some, public-facing confrontation at demonstrations; four participants had been involved with the police in connection with their extremist activity, of whom two received criminal convictions. Given the sensitivity of these histories and the risk of deductive disclosure within a small, publicly contested sample, activity is described here at the level of role and orientation rather than case-by-case detail; more granular, appropriately anonymised description is provided in the doctoral thesis from which this study is drawn.

The sample therefore varied considerably in the nature and severity of involvement. Conviction is not treated here as a proxy for extremist involvement; several participants without convictions had nonetheless been embedded in ideologically extreme milieus for extended periods. This is consistent with definitions of violent extremism that centre on the beliefs and actions supporting such goals rather than on formal criminal outcomes (1, p. 7, citing Canada Centre, 2018).

### 2.4 Data collection

Data collection occurred in two phases. Phase 1 was completed by all ten participants and combined two narrative interviews with structured psychoeducation and collaborative timeline work. Psychoeducation introduced participants to the psychological effects of trauma and adversity, and to basic emotional regulation strategies drawn from Compassion-Focused Therapy (28, pp. 6–41) and Polyvagal Theory (29, pp. 301–318), establishing a baseline of safety before any distressing material was addressed. Timeline work involved co-creating a shared visual record of key life events, ideological involvement, turning points, and existing resources and coping strategies. The opening narrative invitation, used consistently across interviews, was: “*Please tell me your life story in the context of your extremist engagement, from early life through disengagement to the present*.” Follow-up questions were kept minimal throughout, to preserve narrative flow and the participant’s ownership of their account. An additional relational prompt was offered at the end of Phase 1: “*Who could have done what, where, or when to change your trajectory?*” A further small set of reflective questions, including “*Who are you now?”*, was introduced at each participant’s final narrative interview, whether that fell at the end of Phase 1 or Phase 2, to support reflection on identity and change.

Phase 2 offered optional trauma-focused therapy, specifically Eye Movement Desensitization and Reprocessing (EMDR) (30, pp. 1–6), delivered within a trauma-informed, relationally held framework and broadly sequenced according to Herman’s triphasic model of stabilisation, processing, and reintegration (16, pp. 133–154). Six of the ten participants elected to take part, alongside two further narrative interviews. Phase 2 built on the stabilisation already established in Phase 1; a processing phase used EMDR to work through specific unintegrated material identified by participants; and, for some participants, a later phase used ACT to support values-based living and future orientation. These modalities are described here because they shaped the pacing and content of the relational work from which narrative change is analysed, not because the study evaluates their comparative or combined clinical effectiveness. No claim is made that observed changes are attributable to EMDR or ACT specifically rather than to the wider therapeutic relationship, and the analysis does not code for or evaluate technique-specific outcomes.

In practical terms, therapy was delivered individually and remotely, using secure online platforms, which allowed participation across the three continents from which the sample was drawn. Session frequency and total contact varied by participant and clinical need rather than following a fixed protocol, consistent with the study’s trauma-informed, relationally paced design. Across this group, attended sessions ranged from approximately 17 to 29 (mean approximately 22), typically weekly during the earlier stabilisation phase and shifting to fortnightly or three- to four-weekly intervals as work moved into consolidation. Missed or cancelled sessions, recorded as such rather than omitted, reflected a mix of acute practical pressures, such as financial precarity or housing instability, and the emotional difficulty of the work itself, including fear of judgement and fluctuating trust, rather than any single cause.

### 2.5 Emergent conceptual model

The analysis is organised around an emergent conceptual model, constructed retrospectively from participants’ accounts, linking therapeutic engagement to the four capacities examined in this paper. Processing of previously unintegrated distressing material used EMDR, within a relational frame first stabilised through Compassion-Focused Therapy and Polyvagal-informed regulation work. This structured, relationally held work appeared, in participants’ accounts, to be associated with a reduction in affective threat sufficient for participants to remain present with, rather than avoid, distressing content (affect regulation).

As affect became more tolerable, participants described becoming more able to revisit and integrate previously dissociated or shame-laden material into a more continuous self-account (narrative coherence).

As narrative coherence increased, and as the relational context demonstrated consistent, non-judgemental responsiveness over time, participants described becoming more able to experience guidance and support as credible rather than threatening (epistemic trust).

Finally, as epistemic trust and narrative coherence developed, participants described becoming better able to hold responsibility and complexity without collapsing into shame or projection, supporting more flexible moral reasoning and relational conduct (moral agency).

This sequence, regulation, then narrative integration, then epistemic trust, then moral agency, is proposed as a provisional, working order of development. It is broadly consistent with participants’ accounts and with Herman’s (16) triphasic model, but is not presented as a fixed or invariant pathway. The qualitative, non-experimental design cannot establish this sequence causally, and its elements plausibly overlapped and recurred rather than proceeding in a single direction for every participant. Sessions numbers varied, reflecting the study’s trauma-informed commitment to pacing according to participant readiness rather than a fixed protocol. The analysis therefore treats sufficient relational continuity, rather than a specific number of sessions, as the operative condition for change, consistent with participants’ own accounts of what mattered to them. Although presented here, in Methods, before the Results are described, this conceptual model was not specified in advance and then tested against the data. It was not part of the original research design; the sequence became visible only as patterns in participants’ accounts accumulated during analysis, consistent with the inductive approach described in Section 2.6. It is presented before the Results for readability, not because it preceded or shaped the analysis.

### 2.6 Analytic and conceptual framework

Analysis was guided by a narrative analytic approach attentive to both content and form. The analysis traced longitudinal changes in how participants narrated their experiences, focusing on shifts in emotional regulation, identity construction, moral reasoning, and engagement with social, practical, and civic domains.

The analytic framework drew on established psychosocial concepts to support interpretation of change over time. These included narrative identity (31, pp. 233–238), epistemic trust (32, pp. 14–19; 15, pp. 2–5), and trauma-informed models of emotional regulation (28, pp. 9–12). Epistemic trust has also been operationalised and empirically measured in clinical and developmental research, supporting its use as an analytically tractable construct rather than a purely theoretical concept (33, pp. 1–3).

Narrative material was analysed thematically. Polkinghorne distinguishes between analysis of narratives, the identification of common themes across participants’ stories, and narrative analysis, the configuration of disparate data into a single researcher-authored account (34, pp. 5–23). This paper followed the former approach, aligned with Riessman’s thematic tradition (35, pp. 94–133).

Each participant’s account was first read as a whole, then structured into bounded stories using Labov and Waletzky’s account of narrative form, attending to turning points, reversals, and resolutions (36, pp. 3–38). Within and across these stories, thematic tracking then examined what was said explicitly and what remained implicit, who the account appeared to be addressed to, how participants positioned themselves in relation to others, including shifts in the boundary between “us” and “them,” and what function each story appeared to serve, whether justifying, explaining, reinforcing, or reorienting an emerging account of self.

These concepts were selected for their relevance to both the study population and the analytic aims. Narrative approaches were used to allow participants to organise and define their experiences in their own terms rather than through predefined categories, which is particularly important in contexts shaped by trauma and prior loss of control. Trauma-informed models of emotional regulation were drawn on to support interpretation of how participants made sense of distress in non-pathologising ways that emphasised responsibility without shame.

Epistemic trust was not imposed as a prior analytic lens but emerged inductively through engagement with both the literature and participants’ accounts as a salient mechanism shaping openness to support and change. The selection and integration of these concepts reflect the researcher’s clinical training, including expertise in EMDR, and engagement with the relevant literature, while remaining analytically open to the possibility that other psychosocial frameworks could illuminate similar processes from different perspectives.

Change over time was identified by tracking shifts in the narrative dimensions described above, who the account was addressed to, how participants positioned themselves in relation to others, and what function each story appeared to serve, across each participant’s interviews at successive timepoints, alongside a holistic re-reading of each participant’s full narrative arc. Shifts in these narrative dimensions were then interpreted in relation to the four psychosocial capacities examined in this paper: for example, a narrowing of the “us/them” boundary was read as evidence of moral agency and depolarisation, while a shift from an audience oriented toward justification to one oriented toward reflection was read as evidence of narrative coherence. Analytic rigour was supported through several further measures: temporal distance was maintained between data collection and analysis to reduce the influence of immediate clinical impressions; transcripts and emerging interpretations were reviewed by two doctoral colleagues, each independently reviewing two transcripts, with one transcript reviewed by both, covering approximately 10% of the total interview material; and all participants were invited to a follow-up conversation to discuss early thematic impressions, with five choosing to take part. These conversations did not seek to validate findings, but to explore convergence and divergence in how participants and the researcher understood emerging patterns.

Not all participants showed uniform or complete change across every domain, and these cases were analytically informative rather than treated as noise. For P5, P8, and P9 in particular, participants described genuine shifts in emotional regulation and self-understanding while remaining practically or socially “stuck” in other respects. Distinguishing an external constraint on reintegration from an internal capacity that had not fully developed is not always possible from narrative material alone, and this analysis does not claim to resolve that distinction with certainty. Where this interpretation was applied, it was informed by participants explicitly naming external barriers, such as lack of employment, housing instability, or unwelcoming social responses, while also describing concrete examples of changed internal functioning, for example tolerating a distressing memory, or engaging calmly with a person they would previously have avoided, that were not contingent on those external conditions being resolved. This combination was read as evidence that development in the capacities examined here need not stall or reverse under external constraint, even where its consequences were limited.

This reading remains an interpretation rather than a demonstrated finding, and the same accounts could in principle be read as showing partial rather than complete development of these capacities; the distinction matters analytically because the two readings imply different explanations for the same pattern. This pattern is consistent with, and strengthens, the account of reintegration as an ecological process developed in Section 3.4.

### 2.7 Ethical considerations

Ethical approval was obtained from The Open University’s Human Research Ethics Committee. Given the sensitivity of participants’ histories, particular attention was paid to informed consent, confidentiality, and participant wellbeing. Written consent was obtained from eight participants via signed consent forms; in the remaining two cases, oral consent was obtained and audio-recorded, due to technical difficulties returning signed forms.

To manage the dual role, participation in therapy and participation in research were treated as analytically and ethically distinct: separate consent processes were used for each, participants could take part in either or both, and clinical material was excluded from analysis. This separation was designed to address the power imbalance inherent in the dual role, ensuring that participants were never in a position where declining research involvement risked their access to clinical support (37, pp. 217–221). Declining to participate in the research carried no bearing on access to therapy, which remained available in full as it would have been outside the study. In practice, no participant took up therapy without also taking part in the research. Participants could also withdraw from either component independently, at any point, without consequence.

The study was designed to avoid instrumentalising therapeutic engagement for research purposes. Therapeutic work was conducted in accordance with professional ethical standards, and analytic use of data was limited to participants’ narrative interview accounts. Broader ethical questions concerning voluntariness, coercion, and the civic dimensions of reintegration, which extend beyond the immediate research design, are addressed in the Discussion.

## 3 Results

The findings describe how therapeutic engagement supported a set of interrelated psychosocial capacities among participants who had already disengaged from extremist or extremist-adjacent environments. Rather than representing uniform or linear change, these capacities developed unevenly over time and shaped participants’ ability to regulate affect, reconstruct identity, exercise moral agency, and engage with the practical and relational demands of reintegration.

The analysis focuses on recurring mechanisms observable within therapeutic engagement that supported post-exit adjustment, while also attending to variation in how change unfolded across individual trajectories. The sections that follow trace these processes in turn, illustrating how emotional regulation, narrative repair, moral agency, and reintegration were differentially supported and sustained.

Throughout the sections that follow, references to capacities being “supported,” “developed,” or “emerging” describe patterns interpreted from participants’ own accounts, rather than mechanisms this study is positioned to demonstrate. Given the dual-role design discussed in Section 2.2, these patterns should be read as what became visible and interpretable within a sustained, relationally embedded research process, not as independently verified psychological processes. Where a clean, same-theme comparison was available across a participant’s interviews, this is shown directly; elsewhere, quotations illustrate a single point within an analytic process that tracked each participant’s full narrative arc across all interviews, as described in Section 2.6, rather than resting on any single quotation alone.

### 3.1 Emotional safeness and the emergence of regulation

Across participants’ accounts, therapeutic engagement was described as changing how distress was experienced and tolerated, alongside a gradual emergence of emotional safeness and affect regulation. Rather than describing distress as eliminated, participants described becoming able to remain with emotional states that had previously felt overwhelming, without fear of humiliation, retaliation, or abandonment by others.

Participants described becoming better able to slow internal reactions, attend to bodily cues, and stay with distress instead of responding through avoidance or suppression. This shift was often expressed metaphorically. This is illustrated here through paired accounts from the same participant at two points in the study.

Earlier in the study, P4 described a period of acute internal conflict, in which a clear, rational understanding of his situation was repeatedly overridden by an intense and involuntary emotional state:

“My rational mind knew for a fact that I was not possessed … but for some reason … it’s like my animalistic, emotional brain took over and said, ‘Well, what if you are possessed? What if there is something wrong with you?’ even though simultaneously I knew rationally there wasn’t but the emotional side took over.” (P4, N1)

In a later interview, P4 described a markedly different relationship to that same kind of internal state:

“It’s like your mind being … imagine you’ve got a sky that’s really cloudy and you can’t think, and then bit by bit it becomes more visible … the clouds lifting and the thunderstorm calming down.” (P4, N4)

This shift is read here not as a change in P4’s underlying way of experiencing the world, but as a reduction in the degree of internal conflict between his rational understanding and his emotional state, alongside the development of a language and framework through which to describe and hold that experience, consistent with the account of emotional regulation given in Section 1.

For several participants, this shift became more pronounced during the EMDR-focused processing phase, when previously unintegrated memories were revisited directly; this is noted here as contextual information about when change became visible in the narrative record, rather than as evidence that EMDR was the specific mechanism of change, since regulation also continued to develop outside dedicated processing sessions.

P8 described this shift in similar terms, attributing it directly to the EMDR work:

“…I went through a period where I wasn’t leaving the house at all, and I was afraid to leave the house … I think the EMDR was the most helpful piece of it because I was really stuck on many issues and having them cleared from my mind has just been really helpful as far as getting to where I am today.” (P8, N4)

Where participants themselves named EMDR as the salient factor, this is reported as their own account of what stood out to them, rather than as evidence isolating EMDR as the operative mechanism.

Regulation emerged cumulatively through the combination of structured therapeutic containment and the presence of a reliable relational other. Repeated experiences of remaining with distress and discovering it to be survivable appeared to support the development of regulation as a foundational capacity, enabling subsequent reflection and integration.

This pattern is consistent with models describing violent extremism as oscillating around unregulated traumatic activation, in which the capacity to tolerate rather than discharge distress marks a departure from that pattern (38, p. 1). It also stands in contrast to clinical case evidence linking unresolved trauma and dissociation to vulnerability for radicalisation in the first place (39, pp. 1–11): there, dissociation marks susceptibility, its absence here marks a shift toward sustained engagement with distress.

### 3.2 Narrative repair and identity reconstruction

As affect tolerance increased, participants became better able to revisit prior experiences without collapse, supporting the development of more continuous and liveable self-narratives. Changes in emotional regulation were accompanied by shifts in how identity was organised over time, allowing responsibility to be integrated without defensive distancing.

For P4, narrative repair involved maintaining continuity of self while acknowledging past actions:

“I’ve processed what’s happened in my life, taken my old self and my present self and combined them into one whole … that is me.” (P4, N3)

P7 described a similar reworking of self-understanding through the metaphor of an identity map:

“…When I was younger, it would have just said white nationalists and then maybe the heritage or family heritage that I had known about. Nowadays, it’s a bunch of things: introverted, I’m not gonna lie, I am; dirty-minded; thrill seeker; artist; doing this for a living … I can see that I’m capable of both good and bad, like everybody else … But nowadays I’m not the same person as I used to be.” (P7,N4)

For others, identity reconstruction was expressed less through explicit reflection and more through changes in how life was lived, including engagement with work, relationships, and future-oriented roles. Across accounts, narrative repair involved developing the capacity to hold vulnerability and agency simultaneously. As narratives became more coherent, participants increasingly positioned themselves as active authors of their futures, with less emphasis on being defined solely by past experiences.

This mirrors accounts of former combatants’ transition from aggression to recognition, in which renewed capacity for relationship depends on the ability to hold a continuous account of the self that includes past harm without being defined by it (40, pp. 71–89), and is consistent with work identifying identity reconfiguration, rather than simple disavowal of a prior self, as characteristic of exit narratives among former far-right extremists (41, pp. 1–37).

### 3.3 Moral re-evaluation, depolarisation, and relational agency

Improvements in emotional regulation and narrative coherence were closely linked to changes in moral reasoning and reductions in polarised ‘us/them’ thinking. As internal threat became more manageable, participants described greater capacity to attend to responsibility, consequence, and relational impact in everyday interactions.

Moral agency was enacted less through ideological realignment than through practical orientation and everyday decision-making, including boundary-setting and selective withdrawal from contexts experienced as hostile or misaligned. As participants became less organised around internal threat and more able to sustain coherent accounts of self and others, polarised social perceptions softened. This shift is illustrated here through P3’s account of the same theme at two points in the study.

As P3 explained in an earlier interview:

“One of the big things is I can talk to people on the left, where I could never talk to people on the left … If you’d put somebody left wing in front of me, probably before I’d started, I would have felt temper … like a sort of Bloods and Crips sort of attitude … There’s still gonna be things that are emotive with me. But I tend to steer away from them, to be honest.” (P3, N3)

By the final interview, this had developed further:

“But the strange thing is when you go through therapy, you eventually stop seeing it as us and them. I talk to people who are members of the Labour Party and the Green Party now. I would never have done that … As soon as I saw them, I would have probably frowned, and the hatred would have come foremost of my brain.

But now, I’ll stop and speak to them. They’ve just got a different opinion to myself. They’re not hated people … Whereas once you’ve gone through it (therapy), it sort of broadens your perspective and you see … all of the aspects of them as people. You might not agree with them … but it doesn’t mean you have to hate them” (P3, N4)

P5 also located wrongdoing in circumstance rather than character:

“…people do bad things or wrong things or evil things, not because they’re necessarily evil, but because they’re ill-informed … be smart, be strong, and don’t let evil take root.” (P5, N4)

Across accounts, depolarisation was experienced as an affective and relational shift rather than a sudden cognitive change, emerging alongside increased emotional stability and narrative coherence.

This pattern is consistent with work identifying identity transformation, rather than ideological conversion in isolation, as central to how former right-wing extremists narrate their own exit (42, pp. 88–109).

### 3.4 Reintegration as an ecological process

Reintegration marked the point at which emerging psychosocial capacities encountered external social and institutional conditions. While therapeutic engagement supported capacities for regulation, reflection, and agency, the durability of these changes depended on the presence of stability, recognition, and access to meaningful social roles (43, pp. 16–19; 44, pp. 131–159).

Where internal capacity aligned with opportunity, participants described enacting new identities through routine, responsibility, and social participation, including employment, education, and supportive relationships. Where such alignment was absent, material precarity, institutional unreliability, and limited guidance disrupted emerging stability despite psychological gains.

One resource that often helped bridge this gap was peer mentoring: Peer mentoring networks, particularly Life After Hate, often functioned as relational bridges, supporting disclosure, continuity, and practical negotiation of next steps in contexts where formal support was limited or mistrusted. Reintegration was therefore experienced not only as personal recovery, but as a process shaped by social recognition and institutional responsiveness. Overall, post-exit trajectories appeared to be structured by the interaction between developing psychosocial capacity and the relational and institutional environments in which that capacity was tested.

P9 described this tension directly, between internal readiness and external expectation:

“I feel I have options for the first time in a long time that I have options and not having options is huge for me … I struggle sometimes cause I know that there was this thing that was going on with my friends here … It’s putting me in this weird place where people around me think I should do other stuff, but I’m not. Finally, I just stopped thinking about that and just got here. I feel good here … I feel there’s hope for me. I haven’t always felt that, so it’s good.” (P9, N3)

P8 described a related tension, in which financial precarity created a pull back toward extremist-adjacent work specifically because it had once been a viable source of income and a sense of purpose, however harmful:

“Right now, I’m trying to find some more redemptive activities with regard to political organising or other types of redemptive volunteer work … I’ve been trying to get a job, but I’ve also been looking for these volunteer experiences, and it’s not been as easy as I thought it would be. I hate to admit it, but it was much easier to get involved on the right than it was on the left. They were much more willing to immediately embrace me on the right … It’s one of those things where sometimes I feel backed into a corner when I don’t have any money … I would ideally love to have some type of employment that gives me that feeling of redemption … because I feel as though I still somewhat owe a debt to society.” (P8, N1)

## 4 Discussion

This study advances a psychosocial account of disengagement from violent extremism that foregrounds emotional regulation, narrative coherence, epistemic trust, and moral agency as capacities shaping whether reintegration can be sustained over time. Drawing on longitudinal narrative data from former extremists engaged in trauma-informed therapeutic work, the findings suggest that disengagement and reintegration are best understood not as discrete ideological events or compliance outcomes, but as relational and developmental processes of psychosocial capacity-building. This reframing extends existing work that has acknowledged the relevance of mental health in extremism-related trajectories while leaving underlying psychosocial processes under-specified (1, pp. 4–7). The contribution here is not an evaluation of therapeutic effectiveness, but an analytic account of how particular capacities develop, and why they matter for post-exit engagement within real-world social and institutional contexts.

The findings challenge models of disengagement that prioritise ideological change or practical stabilisation in isolation, echoing critiques of risk-led and compliance-focused reintegration frameworks (45, pp. 8–14). Participants entered therapeutic engagement after ideological disengagement had already occurred, yet their accounts indicated ongoing survival-based modes of functioning characterised by emotional dysregulation, epistemic mistrust, and fragmented self-narratives. Participants’ accounts suggest that therapeutic engagement did not initiate disengagement, and for several participants reintegration was already underway before or alongside this study’s therapeutic phase. Rather, therapeutic engagement was associated with the continued development and stabilisation of the psychosocial capacities examined in this paper, consolidating and deepening reintegration already in progress. This is offered as an interpretation of participants’ narratives, not as a demonstrated causal claim. Here, consolidation specifically involved emotional regulation, narrative coherence, and moral agency becoming stable enough to persist beyond the point of exit. It also involved these capacities being tested within, and sustained across, external social, relational, and institutional environments. Viewed through this lens, disengagement and reintegration emerge as processes enacted through participation in everyday roles and relationships, rather than as outcomes secured through ideological realignment or provision alone. This interpretation aligns with narrative approaches that conceptualise identity as an ongoing process of revision and continuity across time.

However, revising a self-narrative is hard to do alone. Across accounts, the restoration of epistemic trust within relationally held therapeutic contexts is proposed here as a salient psychosocial mechanism, consistent with participants’ own accounts of what mattered to them (32, pp. 26–27; 46, pp. 2–5). This study is not positioned to establish it as a demonstrated mechanism, however. Participants’ narratives indicated that emotional safety preceded a sense of being understood, which in turn preceded openness to guidance, support, and alternative ways of living. In conditions of epistemic mistrust, practical interventions were often experienced as misaligned or intrusive, limiting their effectiveness even where material provision was available.

Therapeutic relationships provided a context in which trust could be gradually re-established, enabling participants to engage with practical and social resources in ways that aligned with emerging identities and values. This helps to explain why psychosocial support framed in generic or instrumental terms often struggles to support sustained change when relational conditions are insufficiently addressed. The pattern observed here is consistent with empirical work linking epistemic trust to improved emotion regulation and interpersonal functioning, particularly in contexts shaped by prior threat sensitivity and mistrust (47, pp. 6–8). However, this parallel does not establish that therapeutic engagement caused disengagement or reintegration outcomes, nor that observed changes can be isolated from the effects of time, maturation, or external support. Rather, it specifies a set of psychosocial mechanisms that became visible within a sustained relational context, mechanisms that appeared to shape how participants interpreted, tolerated, and engaged with post-exit demands.

Epistemic trust was not the only capacity to shift in this way. The findings also contribute to debates concerning moral change and depolarisation in the context of extremist disengagement. Participants did not describe moral transformation as the adoption of new ideological frameworks or externally imposed values. Instead, moral agency re-emerged as participants became better able to tolerate emotional complexity and integrate responsibility within more coherent self-narratives. Depolarisation appeared less as a cognitive shift than as an affective and relational one: as internal threat diminished and shame became more manageable, the need to organise social perception around hostility or projection weakened (28, pp. 22–26). From this perspective, polarisation is sustained not only by belief systems, but by emotional and relational dynamics shaped by dysregulation and threat sensitivity. Efforts to counter polarisation through debate or persuasion alone may therefore overlook the psychosocial conditions under which moral complexity and rehumanisation become psychologically viable.

Taken together, these dynamics have implications for how different forms of support are sequenced and taken up within disengagement and reintegration pathways (48, pp. 7–8), so as to maximise impact and avoid overwhelming the client. Analytically, therapeutic engagement appears, in participants’ accounts, to function as an enabling condition, positioned within a broader intervention ecosystem rather than as a corrective response to risk: participants described internal threat reducing and epistemic trust being restored, in ways that appeared to alter how subsequent opportunities for engagement were experienced. As emotional safeness increased and social perception became less organised around danger or mistrust, participants appeared better able to consider guidance, opportunities, and alternative perspectives without immediate defensiveness driven by fear of judgement or failure. Under these conditions, the value of therapeutic engagement lies not only in symptom reduction, but in its capacity to shape how practical, social, and civic interventions such as employment, education, or community participation, are received, interpreted, and taken up as sources of continuity, dignity, and meaning rather than as imposed demands.

This sensitivity to framing has roots earlier in participants’ lives. Participants’ accounts also highlight the importance of how therapeutic support is introduced and framed. For many, adversity had been normalised across the life course, such that experiences of neglect, threat, or instability were not readily recognised as warranting support. Where such experiences were widespread within families or communities, distress was often understood as something to be endured rather than named. This normalisation created a second difficulty: when adversity was perceived as ordinary, struggling in its aftermath was often interpreted as a personal failure. Participants described shame, self-blame, and a sense of being deficient or weak when they found themselves unable to cope in ways they believed others could. Extremist groups were described as temporarily countering these states by offering a sense of worth, agency, and moral significance. In this context, mental health framings that positioned individuals as vulnerable, disordered, or in need of correction risked reactivating precisely the experiences of weakness and devaluation that extremist involvement had helped to defend against. Therapeutic engagement therefore became possible not when support was presented as a response to pathology or risk, but when it was offered as an open, collaborative space for understanding past experiences and present difficulties in ways that preserved dignity and agency.

While therapeutic engagement supported these internal capacities, the durability of change depended on external conditions: stigma, economic precarity, institutional responsiveness, and access to meaningful roles. Participants who described greater continuity over time were typically those able to translate psychological change into everyday contexts that offered dignity, predictability, and recognition; where such alignment was absent, progress risked becoming fragile or stalled despite genuine therapeutic gains. The psychosocial capacities examined in this paper, however they are developed, appear from participants’ accounts to contribute to long-term reintegration: they functioned most effectively when they could be carried into environments capable of sustaining them. Reintegration therefore appeared not solely as an individual task, but as a shared ethical and civic process requiring reciprocity from institutions and communities. Inconsistent, exploitative, or unreliable forms of support risk undermining the relational conditions of trust, openness, and stability on which sustainable change depends. Rather than proposing a specific service model or integrated system of care, the analysis here is concerned with identifying the psychosocial conditions under which support, therapeutic or otherwise, becomes usable, durable, and sustaining. While these findings point toward wider governance and institutional implications, the analysis remains intentionally confined to psychosocial processes observable within therapeutic contexts, to maintain analytic focus.

This analytic focus does not preclude practical relevance, however: understanding these psychosocial processes in detail is itself useful to those working directly with post-exit populations. From a practice perspective, these findings have relevance for non-academic actors and frontline organisations involved in extremism prevention and post-exit support. They help to explain why practical or ideological interventions may falter when psychosocial capacities for regulation, trust, and narrative coherence are insufficiently supported. Consistent with best-practice frameworks for reintegration and return from extremist contexts, trauma-informed approaches emphasise emotional safety, identity development, and continuity of relational support as conditions for sustained engagement (49, pp. 12–15).

These findings suggest several more specific implications for practice. First, behaviour that might be read by practitioners or institutions as resistant, guarded, or non-compliant may sometimes reflect underlying epistemic mistrust or perceived threat rather than a lack of motivation to change. This includes behaviour such as declining to engage with an intervention, withholding information, or appearing evasive. Where this is the case, responding to such behaviour as a compliance problem risks reinforcing precisely the mistrust that produced it. A formulation-based approach that asks what threat or mistrust the behaviour may be protecting against, rather than assuming disengagement or defiance, may be more productive.

Second, establishing psychological safety and epistemic trust may need to precede, or run alongside, the introduction of specific content-based interventions, whether ideological, practical, or therapeutic. Interventions offered before this groundwork is in place may be experienced as intrusive or threatening, regardless of their content. This does not imply a strict sequence in which therapeutic work must always come first, however. The relationship between internal psychosocial capacity and external stability is plausibly reciprocal rather than linear: stable housing, employment, or supportive relationships may themselves support the development of emotional regulation and trust, just as therapeutic work may support engagement with practical support. Practitioners working across different settings and timescales are better placed than this study to judge which starting point best fits an individual’s circumstances.

Importantly, these findings do not suggest that trauma-focused therapy of the kind described here is necessary for everyone disengaging from extremism, nor that its absence precludes successful reintegration. Several participants in this study distinguished clearly between aspects of their thinking that had changed through therapeutic engagement and aspects, particularly political conviction itself, that they did not expect therapy to change and did not experience it as trying to. As one participant put it, reflecting on continuing political disagreements he expected to hold indefinitely: “…it’s gonna be an emotive subject. It’s not going to be cured by psychology” (P3, N3). The value of the psychosocial capacities examined in this paper may lie less in prescribing trauma-focused therapy as a universal requirement, and more in informing individualised assessment and formulation: identifying which capacities appear underdeveloped for a given individual, and using that understanding to inform how support, of whatever kind, is introduced and sequenced.

These implications intersect with existing psychologically informed models of change for terrorist rehabilitation. The Healthy Identity Intervention, for example, integrates risk-need-responsivity principles with attention to identity and the therapeutic relationship in statutory UK settings (50, pp. 63–92). The present findings extend this work by specifying particular relational and psychosocial conditions, epistemic trust and narrative coherence in particular, that may underpin whether such interventions are experienced as responsive rather than imposed.

Crucially, the capacity for therapeutic engagement to function in this way depended in part on whether participation in therapy was experienced as voluntary and dignity-preserving. Where engagement was perceived as pressured or instrumentalised, participants’ accounts and the wider literature suggest that threat, shame, and epistemic mistrust may be reactivated, undermining the conditions required for sustained change.

### 4.1 Ethical considerations revisited: securitisation, voluntariness, and reintegration as a civic process

These findings also raise ethical questions specific to therapeutic work conducted in securitised contexts, where support for former extremists sits close to surveillance, risk management, and public protection imperatives (51, pp. 609–627). The voluntariness this study depended on cannot be assumed elsewhere: even where therapeutic engagement is not formally mandated, it can be shaped by implicit pressure arising from statutory involvement, licence conditions, or a wish to be seen as compliant by services with power over housing, benefits, or legal status. In the present study, participation was structurally voluntary and kept separate from statutory obligation. This cannot be assumed to generalise to therapeutic provision delivered within statutory disengagement programmes, however, where engagement can form part of a condition of licence or comparable order. Where therapeutic engagement is embedded within such securitised and quasi-mandatory arrangements, the dignity-preserving, collaborative framing identified in this study as a condition for change becoming possible may be harder to sustain. Further research is needed on how voluntariness can be meaningfully protected under conditions of implicit or explicit coercion.

This tension bears directly on the mechanism this paper identifies as central to change. If epistemic trust depends on experiencing another person’s guidance and responses as benign rather than threatening, this becomes considerably harder to sustain where that same person, or the service they represent, also contributes to decisions about an individual’s risk classification, licence conditions, or continued liberty. In such settings, disclosures that would support therapeutic work, admitting doubt, describing an unresolved sympathy, acknowledging a lapse, may carry real consequences for the individual’s management. This creates a structural incentive toward exactly the kind of guarded, selectively self-protective account that this study’s design was intended to work against. This does not mean epistemic trust is unattainable in statutory or securitised settings. It suggests, rather, that establishing it may require more explicit and sustained attention to the boundaries between therapeutic and risk-related roles than was necessary in the present study, where that separation was structural rather than something practitioners had to actively maintain within a single service.

As noted above, reintegration already appears in this analysis as a shared civic process rather than a purely individual achievement. That claim carries a further ethical dimension worth making explicit: it implies an obligation on institutions and communities to extend recognition, trust, and opportunity to individuals who, in some cases, caused real harm. This obligation sits alongside legitimate public concerns about accountability, safety, and the interests of those harmed by extremist activity. The present analysis does not resolve this tension. It aims instead to specify the psychosocial conditions under which reintegration becomes possible, while recognising that the civic and moral legitimacy of extending recognition to former extremists remains a matter for wider societal and policy deliberation rather than one this study can settle.

### 4.2 Limitations

Several limitations should be noted. The qualitative design and small sample size preclude claims of statistical generalisability. Participants were self-selected and had already disengaged prior to entering the therapeutic phase, limiting conclusions regarding prevention or early intervention.

A further limitation concerns selection into the therapeutic phase itself: as detailed under Participants, non-entry into therapy was associated with prior completion of trauma-focused therapy elsewhere, a competing acute crisis, or a preference to share one’s story without therapeutic processing. It is therefore plausible that the six participants analysed here were, on average, more stable, better able to sustain regular contact, or more disposed to reflective engagement than those who did not continue into this phase. The findings should be read as describing psychosocial processes that became visible under conditions of relative stability and sustained engagement, rather than as generalisable to the full range of post-exit trajectories, including those characterised by greater instability or more limited access to sustained support.

This selection also bears directly on the mechanism and sequence proposed in this paper. All six participants had already disengaged, remained in therapeutic engagement for a sustained period, and were selected in part for their capacity to do so. The proposed order of development, regulation, then narrative integration, then epistemic trust, then moral agency, should therefore be read as a pattern observed within a group already disposed toward this kind of change, rather than as a sequence this study can show to hold more generally.

The findings should therefore be understood as theory-generating rather than as evaluations of intervention effectiveness. As with any relationally embedded research, participants’ narratives may have been shaped in part by the therapeutic context itself; however, the analysis focuses on patterns of change observable across time rather than attributing causality to specific therapeutic techniques. Future research could examine how similar psychosocial processes unfold across different institutional contexts, explore the timing and dosage of therapeutic engagement, and investigate how relational interventions interact with practical support over longer periods and across diverse governance settings.

## 5 Conclusion

Together, the analysis offers a theoretically grounded, empirically informed account of how therapeutic engagement relates to disengagement from violent extremism and the conditions under which reintegration is sustained. By foregrounding emotional regulation, narrative coherence, epistemic trust, and moral agency, it reframes disengagement and reintegration as processes of psychosocial capacity-building that extend beyond ideological conversion or compliance. Change, from this perspective, emerges through the ongoing negotiation between internal capacity and external possibility within relational contexts capable of holding both vulnerability and responsibility.

## Data Availability

The datasets presented in this article are not publicly available due to the highly sensitive nature of the material and ethical restrictions related to participant confidentiality. Anonymised excerpts may be made available by the corresponding author upon reasonable request, subject to ethical approval and in accordance with participant consent agreements. No identifiable data will be shared. Requests to access the datasets should be directed to deepti.ramaswamy@open.ac.uk.
