# Supplementary material for: Disengagement and reintegration as psychosocial capacity-building after violent extremism
Source: Front Psychiatry. 2026 Oct 16;17:1814567. doi: 10.3389/fpsyt.2026.1814567 (PMC13721798; doi:10.3389/fpsyt.2026.1814567)
Supplement: Supplementary file 1 [file SupplementaryFile1.pdf]

Disengagement and reintegration as psychosocial capacity-building after violent extremism

Deepti Ramaswamy

Department of Psychology, The Open University, Milton Keynes, United Kingdom

Supplementary Information

Procedural details supporting evaluation of the study

This Supplementary Information provides additional procedural detail not included in the main article. It does not constitute a complete account of the broader methodological framework informing the research, which is described in the main article, but elaborates specific elements of interview design, ethical containment, and analytic support.

1. Interview prompts

The use and timing of additional prompts beyond the core narrative invitation varied across participants, consistent with the study's trauma-informed stance. Where clarification supported coherence or safety, questions were used to deepen material already raised; in other cases, questioning was limited to avoid emotional overwhelm. All interviews nonetheless addressed the same core narrative domains, and systematic analysis ensured that findings rested on participants' accounts rather than on uniform questioning.

2. Timeline mapping

The timeline remained under each participant's control throughout. Timeline content was used to guide therapy but was not treated as a separate data source.

3. Stabilisation and ethical containment

Given that revisiting traumatic or morally complex experience can destabilise identity, the study operated under a clear duty of care: participants were supported to leave each session no more vulnerable than when they arrived. Interpretation was deliberately deferred until the narrative phase was complete, reflecting a commitment to narrative protection and epistemic humility.

Phase 1 concluded with explicit re-consent for those who chose to continue participation.

4. Reflexivity and analytic support

The independent readings of transcripts described in the main article were sought for contrast rather than convergence, highlighting how positionality shapes interpretation. Divergences were used to open analytic possibilities rather than to adjudicate correctness.

5. Ethics and confidentiality

All procedures were approved by the Human Research Ethics Committee at The Open University (Approval number: 4314).

As stated in the main article, analytic claims are based exclusively on narrative interview material generated within the research phase; no additional data, analytic claims, or findings are introduced in this Supplementary Information. Given the sensitivity of the material and the potential for indirect identification, no full transcripts, extended narrative extracts, or raw data are included here, consistent with the Data Availability Statement in the main article.

End of Supplementary Information
